# Towards a comprehensive description of relative aortic pressure: insights from 4D flow CMR

**DOI:** 10.1186/1532-429X-15-S1-P243

**Published:** 2013-01-30

**Authors:** Alex Pitcher, Pablo Lamata, Sebastian B Krittian, David A Nordslettern, Malenka M Bissell, Jane M Francis, Thomas E Cassar, Alex J Barker, Michael Markl, Stefan Neubauer, Nic Smith

**Affiliations:** 1 Oxford Centre for Clinical Magnetic Resonance Research, Department of Cardiovascular Medicine, University of Oxford, Oxford, UK; 2 Department of Computer Science, University of Oxford, Oxford, UK; 3 Department of Biomedical engineering, Division of Imaging Sciences, The Rayne Institute, King's College School of Medicine, Oxford, UK; 4 Departments of Radiology and Biomedical Engineering, Northwestern University Feinberg School of Medicine, Oxford, UK

## Background

A complete description of the relative spatiotemporal pressure gradients which drive blood flow has been a central goal of hemodynamics research over six decades. We have previously described a novel computational method for the in vivo estimation of these relative pressure gradients in the human cardiovascular system based on 4D flow Cardiovascular Magnetic Resonance (CMR) datasets (reference 1). We now describe the application of this method to allow a comprehensive assessment of the spatiotemporal distribution of relative pressure in the human aorta, based on 4D flow CMR datasets, in both healthy subjects, and in patients with established aortic disease. We have extended the approach by isolating, and individually analysing, the three major components of relative pressure, providing unique insights into the nature and timing of intra-aortic relative pressure changes.

## Methods

**Figure 1:**
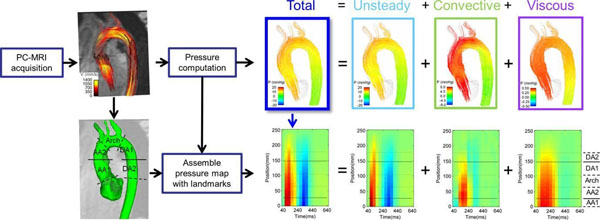
Methodology for the computation of the spatiotemporal maps of pressure in the aorta. The top of the illustration represents the data workflow from a single frame, frame 4, which constitutes the fourth column in the spatiotemporal map, as indicated by the blue arrow. The horizontal lines in the spatiotemporal map correspond to specific plane locations in the aorta, which are defined at the level of the pulmonary artery (the two continuous lines), before and after the great vessels of the arch, and the descending in line with the mitral annulus. These planes divide the aortic anatomy in regions: the ascending aorta (AA1, AA2), the arch and descending aorta (DA1, DA2).

Six subjects underwent time-resolved, phase contrast CMR with 3-directional velocity encoding (4D flow) at 3 Tesla. Three were healthy volunteers and three were patients with established aortic disease (bicuspid aortic valve with associated ascending aortic aneurysm, Type A aortic dissection and Marfan syndrome). Spatiotemporal pressure maps were computed from the CMR flow fields using a finite-element implementation of the pressure Poisson equations. Using this formulation, the individual components of pressure were separated as time-varying inertial ("unsteady"), spatially-varying inertial ( "convective") and viscous component (Figure 1).

## Results

**Figure 2:**
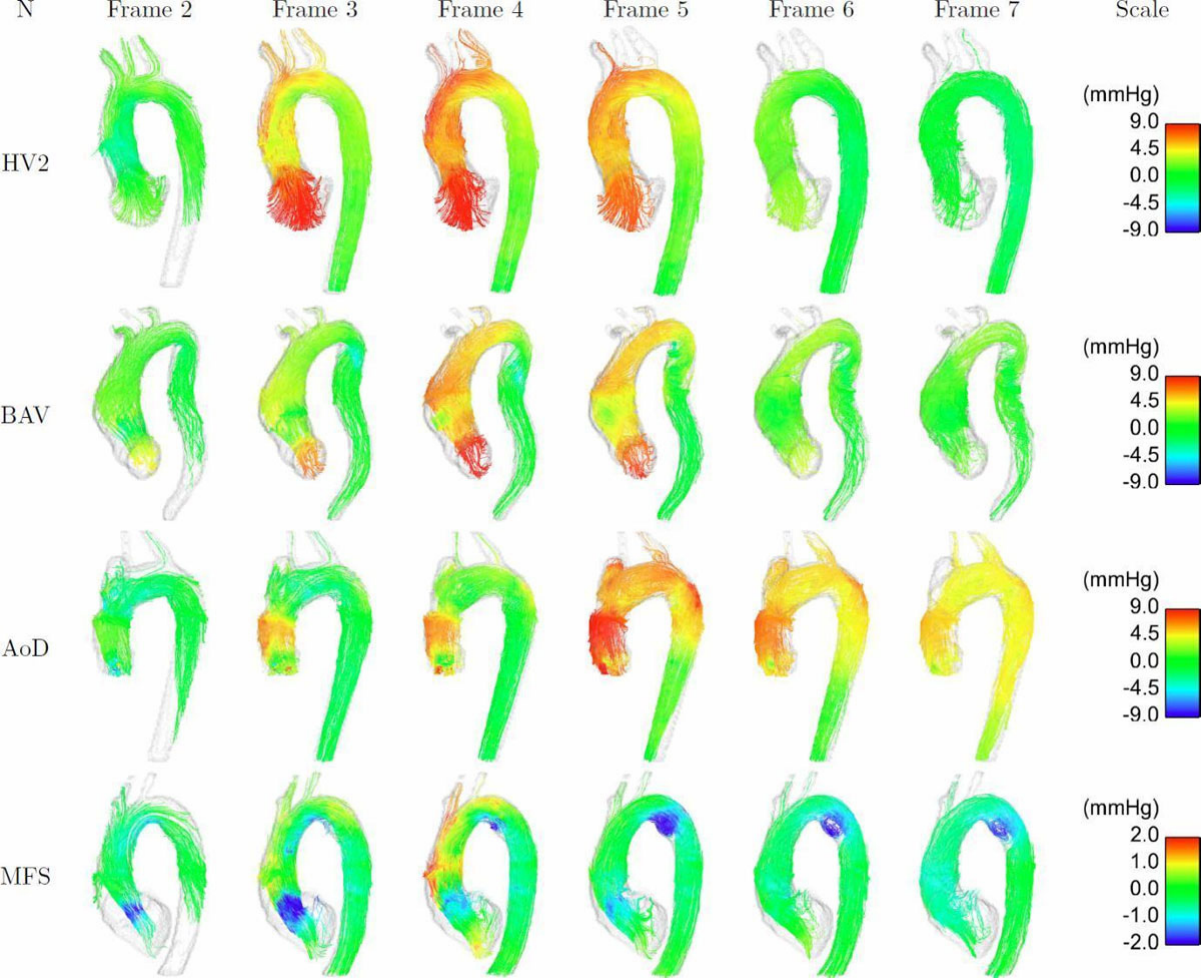
Anatomical inspection of the convective pressure, showing velocity streamlines coloured by pressure, in a healthy volunteer (HV2), a patient with Marfan syndrome (MFS), a patient with an aortic dissection (AoD), and a patient with bicuspid aortic valve, ascending aortic dilation, and a pseudo-coarctation (BAV). MFS and BAV both show vortexes characterised by an abrupt drop of pressure, at the beginning of the proximal descending aorta. AoD shows increased pressure in the aortic root graft, the (dissected) carotid artery, and in the proximal descending aorta at the outer curvature.

Aortic pressure differences are mainly caused by the unsteady effects (15mmHg at instant of peak acceleration), followed by the convective and a small viscous contribution (3.14mmHg and 0.2mmHg respectively at instant of peak velocity). Visualisation of relative pressure maps allowed identification of well-localised abrupt changes in pressure identified in each of the diseased cases. These regions of abrupt pressure difference were explained by either differences in aortic geometry, such as the presence of an aneurysm, a pseudo-coarctation, or the inlet of a dissection, or by complex flow features, such as vortical flow, particularly in the case of convective component of pressure (Figure 2).

## Conclusions

We describe the time-resolved relative pressure distribution, in healthy subjects, and in those with aortic diseases characterised by aortic dilation, demonstrating that relative pressure distributions are consistent in the healthy aorta but differ in disease. The isolation and separate evaluation of the three components of relative pressure provides further unique insights into the timings and contributions of each component to overall pressure differences, with implications for understanding mechanisms of aortic disease in populations and in individuals, and with potential for guiding choice of therapy in future.

## Funding

Alex Pitcher and Pablo Lamata contributed equally to this work. Funding support is acknowledged from the European Community's Seventh Framework Program; the Department of Health via the National Institute for Health Research (NIHR) comprehensive Biomedical Research Centre award to Guy's & St Thomas' NHS Foundation Trust in partnership with King's College London and King's College Hospital NHS Foundation Trust (Lamata, Krittian, Nordsletten, Smith), the British Heart Foundation (Pitcher, Bissell), the Oxford NIHR Biomedical Research Centre (Pitcher, Neubauer), and the Oxfordshire Health Services Research Funds (Pitcher, Cassar). Stefan Neubauer and Nic Smith acknowledge support from the BHF Centres of Research Excellence at Oxford and Kings College London respectively.
